# Polyethylene-Based Knee Spacer for Infection Control: Design Concept and Pre-Clinical In Vitro Validations

**DOI:** 10.3390/polym12102334

**Published:** 2020-10-13

**Authors:** Yuhan Chang, Mel S. Lee, Jiann-Jong Liau, Yu-Liang Liu, Wen-Chuan Chen, Steve W. N. Ueng

**Affiliations:** 1 Department of Orthopedic Surgery, Chang Gung Memorial Hospital, Linkou 33305, Taiwan; yhchang@cloud.cgmh.org.tw; 2 Bone and Joint Research Center, Chang Gung Memorial Hospital, Linkou 33305, Taiwan; 3 College of Medicine, Chang Gung University, Taoyuan 33302, Taiwan; 4 Department of Orthopedic Surgery, Chang Gung Memorial Hospital, Kaohsiung 83301, Taiwan; mellee@adm.cgmh.org.tw; 5 Research & Development Department, United Orthopedic Corporation, Hsinchu 30075, Taiwan; jjliau@uoc.com.tw (J.-J.L.); Matt.Liu@uoc.com.tw (Y.-L.L.); kevin.chen@uoc.com.tw (W.-C.C.)

**Keywords:** periprosthetic joint infection, two-stage exchange arthroplasty, knee spacer, polyethylene, cement, fatigue, antibiotic elution, wear

## Abstract

Antibiotic-loaded polymethyl methacrylate (PMMA) has been widely applied in the treatment of knee periprosthetic joint infections. However, problems with antibiotic-loaded PMMA-based spacers, such as structural fracture and implant dislocation, remain unresolved. A novel polyethylene-based spacer, designed with an ultra-congruent articulating surface and multiple fenestrations, was introduced in the current study. Validation tests for biomechanical safety, wear performance, and efficacy of antibiotic cement were reported. During cycle fatigue testing, no tibial spacer failures were observed, and less wear debris generation was reported compared to commercial PMMA-based spacers. The volumetric wear of the novel spacer was within the safety threshold for osteolysis-free volumetric wear. An effective infection control was demonstrated despite the application of lesser antibiotic cement in the 30-day antibiotic elution test. The tube dilution test confirmed adequate inhibitory capabilities against pathogens with the loaded antibiotic option utilized in the current study. The novel polyethylene-based knee spacer may offer sufficient biomechanical safety and serve as an adequate carrier of antibiotic-loaded cement for infection control. Further clinical trials shall be conducted for more comprehensive validation of the novel spacer for practical application.

## 1. Introduction

Ultra-high molecular polyethylene (UHMWPE) and polymethyl methacrylate (PMMA)-based bone cement are polymers commonly utilized in joint arthroplasty surgeries. Bone cement has generally been utilized for gap filling or reinforcement of an osteoporotic structure, while UHMWPE has been widely applied in soft bearing components for artificial joint articulation. Total knee arthroplasty (TKA) is currently accepted as the most efficient and successful surgical strategy for the treatment of intracapsular disease of the knee joint, greatly enhancing patients’ quality of life [1,2]. However, postoperative infections remain one of the most devastating and challenging complications after joint replacement, reporting a prevalence of 0.66–3% with 20.3% reported cases requiring revision surgery [3,4,5]. Two-stage exchange arthroplasty, which involves the removal of infected prostheses and introduction of a temporary antibiotic-loaded cement spacers at the infection site, has been widely accepted among treatment options [3,6,7,8]. Antibiotic-loaded cement spacers facilitate in maintaining joint space, limb length, soft tissue tension, and lengthening the period of effective antibiotic release until infection control has been accomplished. The published data supports the utilization of an antibiotic-loaded cement spacer, with reported success rates of 91–96% [9,10]. A systematic review demonstrating the utilization of an articulating or static cement spacer for the preservation of joint mobility resulted in similar success rates for infection control with articulating (or “endoskeleton-type”) spacers providing knee motion to a certain degree [11,12,13,14].

Although articulating knee spacers have demonstrated advantages in joint mobility and clinically successful rates in infection control, issues relating to biomechanical safety contributed by cement material characteristics should be carefully noted. Articulating knee spacer-related complications include squeaking during articulation [15], intra-articular spacer dislocation [12,16,17], and spacer fracture [16,17,18] were reported in previous clinical studies with the utilization of custom-made or commercial articulating spacers. Commercial knee spacers were shown to provide adequate articulating surface for joint movement but lacked sufficient control in deeper, intramedullary canal infections. Surgeons had to implement alternative methods for the construction of an intramedullary spacer to provide sufficient infection control for deeper infection sites by wrapping antibiotic-loaded cement onto spinal rods or Kirschner wires, or by manually shaping the canal spacers [17,19], which could cause surgical inconveniences. In addition, preformed commercial antibiotic-loaded knee spacers do not offer flexible antibiotic options. Efficient infection control may be lacking as a result of inappropriate antibiotic formulations.

A novel polyethylene-based knee spacer, for the purpose of infection control, was developed to enhance the biomechanical safety and surgical convenience of articulating knee spacers. As an antibiotic-loaded cement carrier, the scaffold-like structures within the polyethylene component facilitate cement deposition with antibiotic-releasing properties, while a standardized metallic canal rod is prepared for intramedullary infection control. The current pre-clinical validation study has been designed to confirm the safety and efficacy of the novel device in infection control by conducting the required in vitro biomechanical fatigue, wear, and antibiotic elution testing before approval in clinical applications.

## 2. Materials and Methods

### 2.1. Device Description and Design Concept

**Figure 1:**
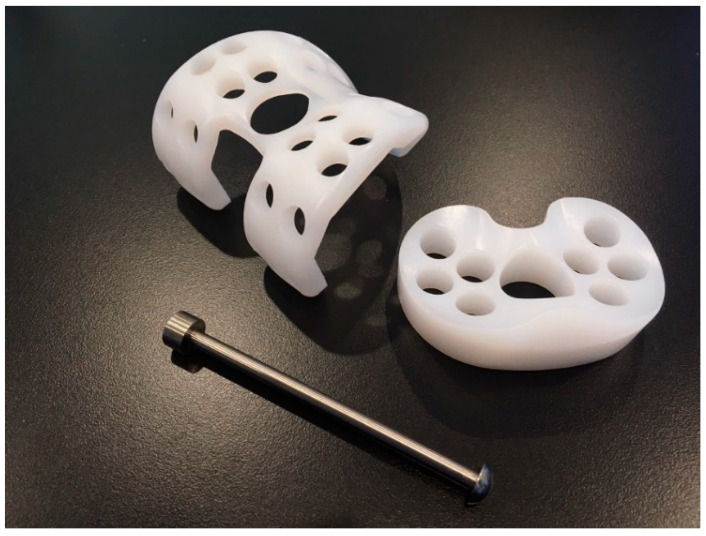
The novel polyethylene-based knee spacer set with canal rod.

The novel spacer set consists of a femoral spacer, a tibial spacer and a canal rod (Figure 1). The spacers are made of medical-grade UHMWPE with verified biocompatibility. The geometrical design of the femoral spacer is based on the commercial cruciate-retaining femoral component (U2 Knee system, United Orthopedic Corporation, New Taipei City, Taiwan), while the tibial spacer is a modification of the ultra-congruent tibial insert design (XUC insert, U2 Knee system, United Orthopedic Corporation, New Taipei City, Taiwan) to facilitate a high articulating congruency with the femoral spacer and better stability [20,21]. The raised anterior edge and posterior lip are designed to reduce the risk of spacer dislocation. Both femoral and tibial spacers are designed with scaffold-like features with the preparation of cylindrical fenestrations on the components. Five femoral and 5 tibial spacer sizes have been prepared for different anatomical demands with anteroposterior (AP) and mediolateral (ML) dimensions shown in Table 1. The hole sizes from size #1 to size #5 components are, respectively, 7.0, 7.5, 8.5, 9.0, and 9.5 mm, enlarging with the increase of size. The number of holes are identical among sizes (25 holes on femoral spacer and 9 holes on tibial spacer, including the larger central fenestration). For safety and feasibility in mechanical machining, at least 1.5 mm polyethylene thickness are spared between holes to avoid possible material discontinuity or insufficient mechanical strength. Holes are regularly distributed on the component while holes located at the inflection region are avoided for mechanical safety. Larger fenestrations have not been positioned at the distal surface center of the femoral spacer and the center of the tibial spacer for operational access to the intramedullary canal, if required. The articulation surface roughness of the spacers has been manufactured to a maximum Ra of 1.5–2 for better anti-wear and smoother articulation. The scaffold-like features on the spacer set implies that a lower cement volume is required to fill the spacers.

**Figure 2:**
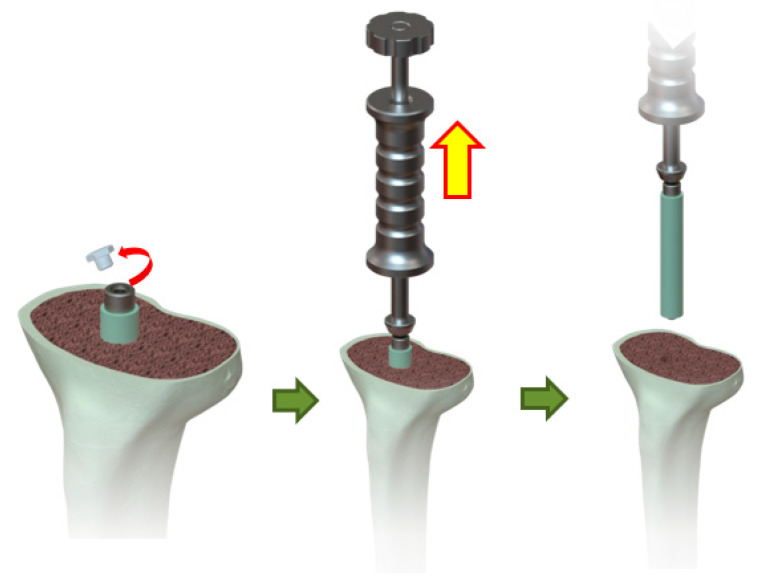
Illustration for the usage of sliding hammer to secure the head of canal rod, and punch reversely to remove the canal rod from the intramedullary canal.

The canal rod is made of a titanium alloy consisting of a 4 mm shaft, a proximal instrument adapter to facilitate the removal of the rod from the canal, and a distal umbrella-shaped hook to ensure distal support of the wrapped cement to facilitate the clean removal of the structure from the canal when infection control has been successfully accomplished (Figure 2).

### 2.2. Preparation of Test Specimens

**Figure 3:**
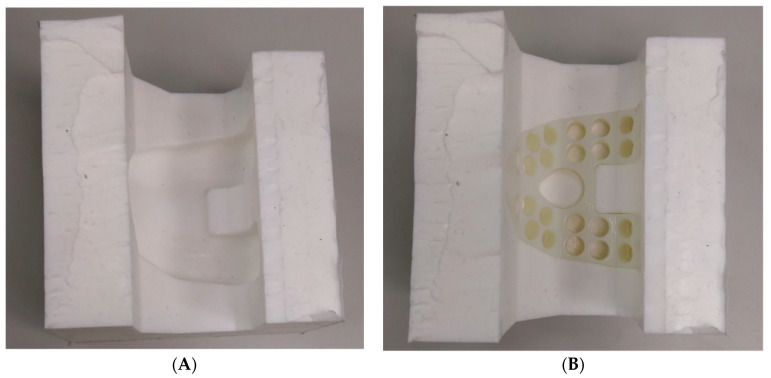
(**A**) The silicon mold; (**B**) Spacers were positioned into the mold for cement filling of the fenestrations.

**Figure 4:**
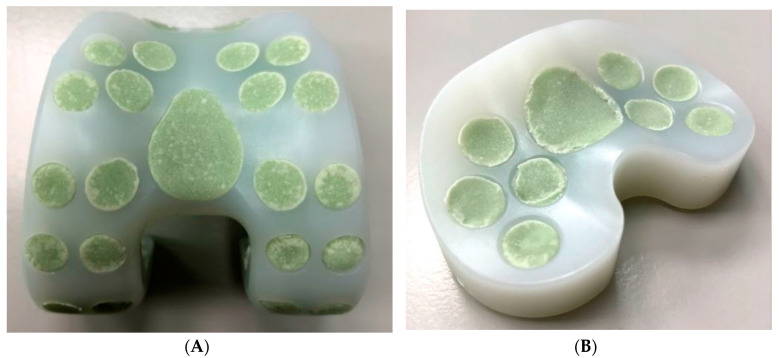
Spacer fenestrations filled by cement: (**A**) Femoral spacer; (**B**) Tibial spacer.

**Figure 5:**
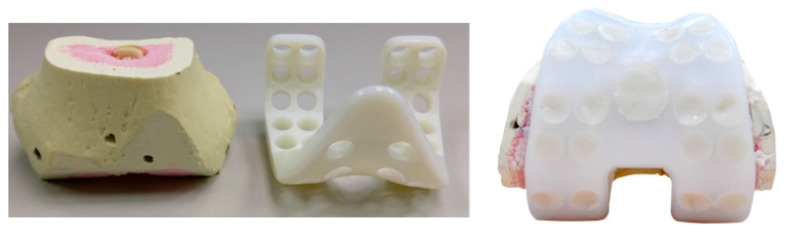

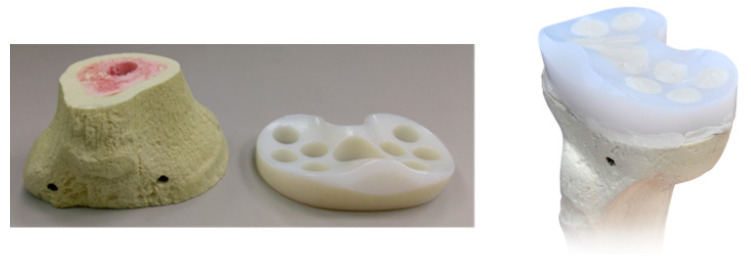
Preparation of (**top**) femoral and (**bottom**) tibial spacers with sawbones. Spacers are attached onto the resection surface with cement.

Size #1 femoral and tibial spacers were selected based on the worst-case principle (the least space spared for antibiotic-loaded cement volume among the 5 sizes) and the available dimensions of the comparative commercial spacer. Antibiotic-loaded bone cement (PALACOS R+G, Heraues, 0.5 g Gentamycin/40 g) was applied to validate the usage of commercial cement in infection control, while regular (non-antibiotic) bone cement (Simplex P, Stryker Orthopaedics, Kalamazoo, MI, USA) was utilized as a cement baseline for antibiotic elution testing. The femoral and tibial spacers were positioned inside customized silicone molds to ensure smooth articulating surfaces during cement preparation (Figure 3). The cement was prepared to a doughy consistency before its manual deposition into the fenestrations until it was fully flushed to the surface of the spacers (Figure 4). No damage or deformation was observed on the UHMWPE spacers during the cement hardening process. Femoral and tibial sawbone (#1121-5-2 & #1117-5, Pacific Research Laboratory Inc., Vashon, WA, USA) specimens, which were prepared for antibiotic elution test, were pre-cut and pressed against cement until full polymerization was achieved (Figure 5). Five antibiotic-loaded tibial spacers were prepared for fatigue testing, 8 sets of antibiotic-loaded femoral/tibial spacers were prepared for wear testing, and 6 sets of femoral/tibial spacers (3 sets with antibiotic-loaded cement and 3 sets with non-antibiotic cement), were prepared for antibiotic elution testing. All specimens were cleaned, sterilized with ethylene oxide, and well packaged according to the product standard operation procedure of the United Orthopedic Corporation, Taiwan.

### 2.3. Tibial Spacer Fatigue Test

**Figure 6:**
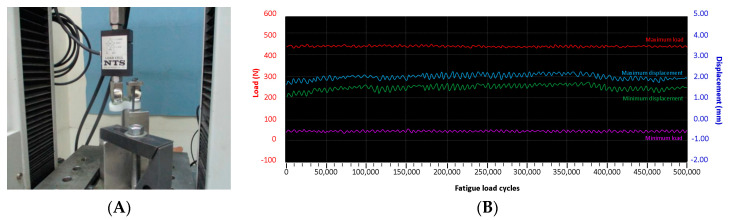
(**A**) Setup of material testing system for tibial spacer fatigue test; (**B**) Recorded load and displacement information.

Fatigue testing was set in accordance with the guidance in ASTM F1800 (Figure 6A). Both sides of the tibial condyles were engaged with a commercial metallic femoral condyle (U2 knee, United Orthopedic Corporation, Taiwan), with one side clamped to the bottom fixture and the other side acting as a cantilever beam load. The suggested guidance criteria for maximal load was at 900 N. However, the knee spacer is categorized as a temporary device with partial weight bearing conditions during its practical use [22]. Therefore, the magnitude of the sinusoidal cyclic load applied was reduced to 50%, at 45 N–450 N, for fatigue testing. The acceptance criterion was determined as “no visible fracture of tibial spacer found after 5 × 10^5^ cycles”, to mimic the maximum 180-day implantation period for general infection control [23]. The test frequency was set at 5 Hz, according to the guidance criteria. Peak and valley displacements were recorded to check if the test specimen was stable during cyclic loading (Figure 6B).

### 2.4. Wear Test

The wear test was conducted according to the guidance in ISO 14243-1. The loading magnitudes were reduced to 50% to account for partial weight bearing conditions and the regular 180-day usage of the knee spacer [22]. Hence, the articulating cycles, which were defined within the guidance, were reduced to 5 × 10^5^ cycles [23], as described in a previous biomechanical study by Mueller et al. [24]. Six sets of femoral/tibial spacers were utilized for testing under wear conditions, while the other 2 sets were prepared as test controls (specimens were pre-soaked only, no wear loads were applied). The dry weight of all specimens was recorded before testing commenced.

**Figure 7:**
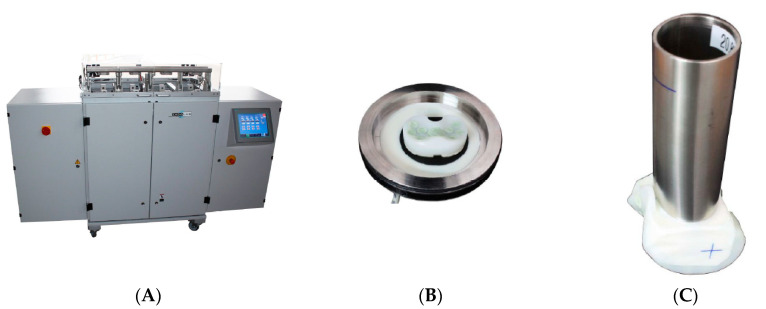
(**A**) The multiple station knee simulator (Endolab), with mounted (**B**) Tibial spacer and (**C**) Femoral spacer for wear test.

All specimens were pre-soaked in the test fluid (calf serum). The specimens were then mounted onto the knee simulator (Endolab^®^ knee simulator, Endolab Mechanical Engineering GmbH, Riedering, Germany, Figure 7), to reproduce implant wear patterns, for wear testing with the modified load conditions (Table 2). Then, 5 × 10^5^ cycles were conducted with loading frequency set at 1 Hz for all 6 specimen sets. All specimens were then dried in a vacuum chamber (37 °C) to facilitate the quantification of the material loss due to spacer or cement wear. The results were compared to published data on commercial knee spacers to confirm the wear safety volume [24]. Test fluids were collected and stirred for approximately 2 min before extracting 2 mL of sample for further testing. Hydrochloric acid was added as the method described in previous literature to dissolve biological particles such as proteins within the specimen [25]. The fluid was then filtered through a 0.05 μm polycarbonate filter using a vacuum pump (Vacuubrand, RZ 5). The filters were then sputter coated with silver to characterize wear particles in the scanning electron microscope (SEM, Leo GmbH, 1530 VP, Bayern, Germany). Five SEM pictures (per filter) were analyzed for particle equivalent circle area diameter, form factor, maximal/minimal ferret diameter, particle area, perimeter, and aspect ratio.

### 2.5. Antibiotic Elution Test 

**Figure 8:**
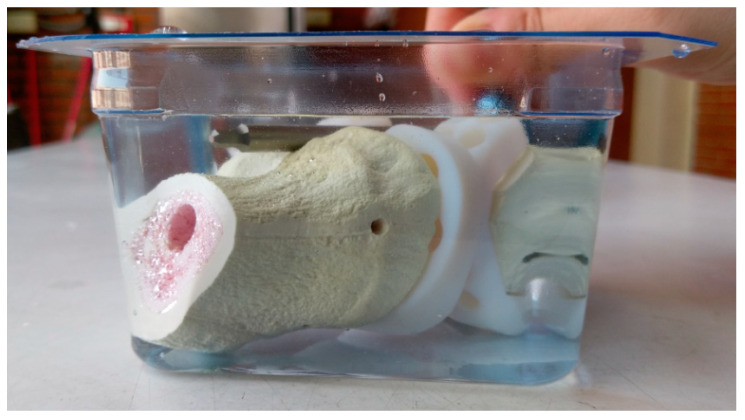
Antibiotic elution test specimens immersed in PBS.

The antibiotic elution test were conducted on the basis of the methods in previous studies [26,27]. The antibiotic-loaded specimens (with sawbone) and non-antibiotic loaded specimens (control group) were immersed in 350 mL of phosphate buffer solution (PBS, pH 7.3) in a 37 °C incubator to mimic the in vivo elution condition in humans for 30 days (Figure 8) [28,29,30]. PBS from each test set was retrieved each consecutive day for the analysis of gentamicin concentrations and bioassay to validate the bacterial inhibitory capabilities of the antibiotic-loaded cement. The retrieved PBS samples were subsequently stored at −80 °C for further analyses. Antibiotic concentration elution assay involved the measurement of gentamicin concentrations in all samples. The acceptance criterion for effective antibiotic concentration should be greater than 1μg/mL, as reported in a previous study [29].

The retrieved 2 mL elution samples were measured for antibiotic activities with the tube dilution method. Bacteria strains, including methicillin-sensitive *Staphylococcus aureus* (MSSA) (ATCC 25923), methicillin-resistant *Staphylococcus aureus* (MRSA) (ATCC 43300), coagulase-negative *Staphylococci* (CoNS) (ATCC 14990), *Pseudomonas aeruginosa* (*P. aeruginosa*) (ATCC 27853), and *Escherichia coli* (*E. coli*) (ATCC 25922) were selected as the pathogens. The samples were added to a tube with 105 bacterial colony forming units (CFUs)/mL in 96-well cultured dishes and incubated at 37 °C for 24 h. Visual identification of bacterial growth was used as qualitative evidence of bacterial inhibition. Clear test fluids were an indication of sufficient eluted antibiotic concentrations. A positive control (without antibiotics) and standard samples with varying antibiotic concentrations were prepared for visual comparison.

## 3. Results

### 3.1. Tibial Spacer Fatigue Test

**Figure 9:**
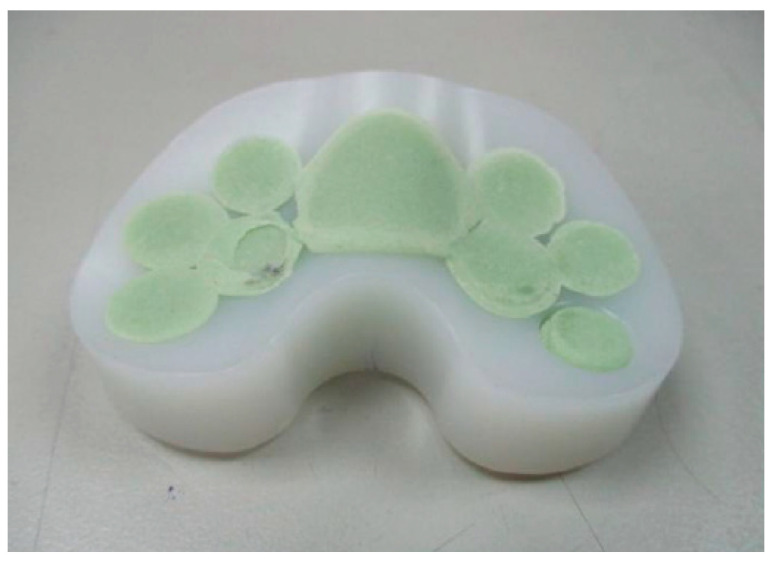
Minor cement cracks found on one of the tibial spacers after fatigue testing.

No visible fracture was found on the tibial spacer after the completion of 5 × 10^5^ fatigue load cycles. The maximal displacement of material remained stable without sudden raise/drop of values recorded by the material testing system. However, one test specimen showed minor cracks on the cement region (Figure 9). No deformation was observed at the polyethylene region.

### 3.2. Wear Test

#### 3.2.1. Wear Rate—Loss of Specimen Weight

Table 3 shows the wear rate by calculating the weight difference of the specimen before and after wear testing. The femoral spacer in specimen #4 was removed from the final averaged performance of the current spacer set as it reported an increase in weight after wear test. The average weight loss of the 5 femoral spacers, excluding specimen #4, was 34.34 ± 14.25 mg, while the tibial spacers had an average weight loss of 18.78 ± 14.46. The wear result of the previously studied commercial spacers is listed in Table 3. 

#### 3.2.2. Morphology of Wear Particles

**Figure 10:**
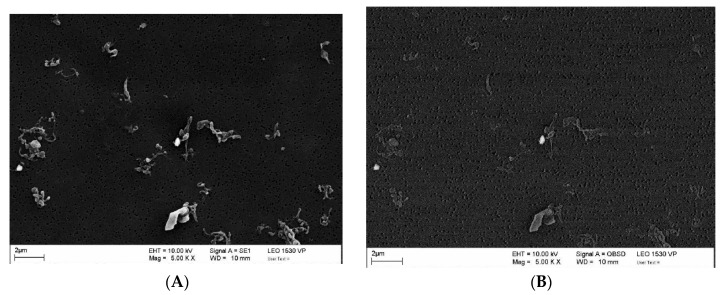
Typical pattern of wear particles: (**A**) Ultra-high molecular polyethylene (UHMWPE) particle with elongated, irregular geometries. (**B**) Cement particle with rounded, regular geometries.

Table 4 represents the morphological characteristics of wear particles, whereas the SEM images (Figure 10) shows the typical morphology of the UHMWPE and cement particles in the current study. In general, the UHMWPE particles were larger than cement particles according to the equivalent circle diameter, maximal/minimal feret diameter, and area of the particles. The greater form factor and lower perimeter and aspect ratio of the cement particles represented a more regular and closely rounded-shaped morphology compared to the UHMWPE particles. Quantitatively, most wear debris originated from the UHMWPE component, and only a small amount of cement particles (within 4 particles) were observed in each of the SEM image. No cement particles were observed in specimen set #5.

### 3.3. Antibiotic Elution Test

#### 3.3.1. Antibiotic Concentration Elution Bioassay

**Figure 11:**
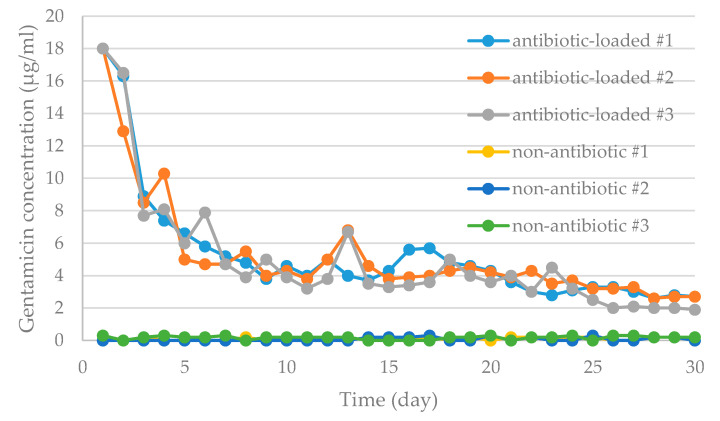
Graphs showing daily antibiotic released from specimens loaded with (antibiotic-loaded #1, 2, 3) or without antibiotic (non-antibiotic loaded #1, 2, 3) in broth elution assay for 30 days. The data are presented as the daily release of gentamicin from specimens with different preparations.

The spacer group (with antibiotic-loaded cement) revealed high elution behaviors at the beginning (Day-1, maximal 18 μg/mL) which gradually decreased with time (Figure 11). The overall elution gentamicin concentration during the 30-day elution period was greater than 1.9 μg/mL, which fulfilled the minimum thresholds for clinical effective antibiotic concentrations (>1 μg/mL) [29]. The control spacer group (with non-antibiotic cement) revealed little to no elution (<0.3 μg/mL) throughout the test period.

#### 3.3.2. Bioassay of Antibiotic Activity

**Figure 12:**
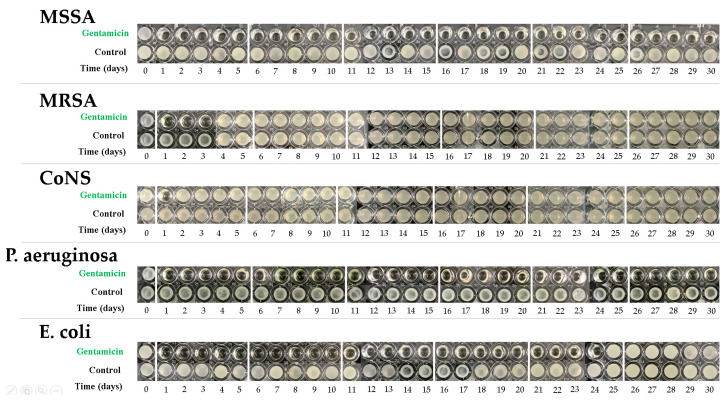
Antibacterial activities against test bacteria in broth elution samples from the antibiotic-loaded bone cement specimens over a 30-day elution period. The wells that appear cloudy indicate bacterial growth.

Figure 12 represents the results of the tube dilution tests for various pathogens. Elution samples performed well in the inhibition of MSSA and *P. aeruginosa* throughout a 30-day testing period with observations of clear tube samples. Antibiotic activities lasted for 24 days in *E. coli* samples, and 3 days in MRSA samples. However, the effective bacterial inhibition of CoNS samples only lasted for one day.

## 4. Discussion

Polymer materials have been widely applied in orthopedic surgery for the purpose of implantation or supplementation. The novel polyethylene-based knee spacer is the first design concept using scaffold-like UHMWPE structures filled with antibiotic–loaded PMMA-based bone cement, to facilitate smoother articulation and control infection of the knee joint. The current study conducted several in vitro pre-clinical validation tests to validate the safety and efficiency of the spacer set. The advantageous material characteristics of UHMWPE supported the fatigue safety of the tibial spacer and ideal anti-wear performance for spacer longevity in simulated knee motion while ensuring sufficient antibiotic elution performance.

Safety is always the key issue in orthopedic implants. Commercial preformed knee PMMA cement spacers or hand-made cement spacers have been widely used in two-stage exchange arthroplasty. However, spacer dislocation and fracture are common complications. Haddad et al. [12] reported 4 cases (N = 4/45, 8.8%) of intra-articular spacer dislocation with the use of commercial preformed spacers. Gooding et al. [16] reported 2 cases (N = 2/110, 1.8%) of dislocations and 3 cases (N = 3/110, 2.7%) of spacer fractures with the same commercial spacers. Spacer fractures were also observed in van Thiel et al. [18] with the use of commercial cement spacer molds. Struelens et al. [17] documented 4 cases (3%) of spacer dislocation, 7 cases (5%) of spacer fracture, and 6 cases of knee joint subluxation between stages with the use of the same commercial molds. Prolonged treatment, multiple surgical procedures, and the increased cost of medical treatments are all associated with unsuccessful infection control [31,32]. Ultra-congruent designs in tibial spacers was adopted in the current design concept because of its ability to enhance stability in articulation and reduce dislocation rates, and successful clinical application in TKA [20,21]. The scaffold-like UHMWPE spacer with fenestrations, introduced in this current study, may facilitate the deposition of antibiotic-load cement, and provide better mechanical flexibility and durability than pure cement, which are weak against bending and shearing loads [33]. The current study performed fatigue testing, with an extreme cantilever load condition to simulate severe bone loss beneath the tibial spacer, on the novel spacer and demonstrated implant survivability without observations of spacer fracture. This observation shows the important role of the UHMWPE in protecting the antibiotic-loaded cement. Surgeons may choose specific antibiotic to target specific bacterial infections by preparing standard non-antibiotic cement with the addition of antibiotics. However, this procedure jeopardizes the mechanical strength of the polymerized cement [34,35], especially when higher antibiotic dosage is required [36]. The current spacer design may ideally resolve this problem as the spacer structure provides a protective mechanism against cement load stresses.

**Figure 13:**
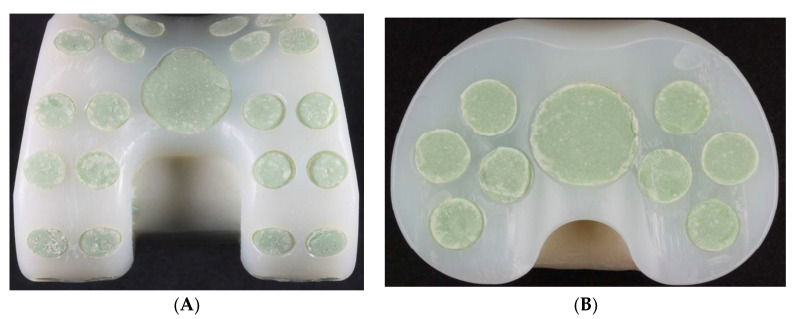
Scratches on UHMWPE spacer components. (**A**) Femoral spacer; (**B**) Tibial spacer.

Component wear at the articulating surface remains an issue as there are no existing surface treatment and there remains uncontrollable complexity within the joint capsular. In addition to structural damage due to surface wear, polymer and cement wear debris morphology [37,38,39,40] and quantity (volumetric wear) [41] are associated with the prevalence of osteolysis. Wear patterns on the spacer surfaces after wear testing is observed in Figure 13, and wear scratches were observed at the UHMWPE surface. Further SEM examination observed few cement particles (Figure 10, Table 4). However, morphological complications in general joint prosthesis designs are more difficult to overcome due to material characteristics and the scale of articulation. Hence, the reduction of wear debris to avoid osteolysis is a critical consideration. Elke and Rieker [41] reported that the estimated threshold for osteolysis-free volumetric wear is around 670 mm^3^. According to the wear testing results in the current study, the dry weight loss of the polyethylene-based spacers was less than that of the commercial cement spacers (Femur: 34.34 mg vs. 149.6 mg; Tibial: 18.78 mg vs. 226.0 mg) [24]. The volumetric wear of the novel spacer set in the current study, by calculating the average wear volume by loss of dry weight and density of the UHMWPE material (ρ = around 0.95 mg/mm^3^), was only 55.92 mm^3^, lower than the reported osteolysis-free volumetric wear threshold. The ideal surface condition of UHMWPE spacers (maximum Ra, 1.5-2), which may have contributed to the volumetric wear results, demonstrated better anti-wear performance than commercial knee spacer with full-cement coverage.

Engineering drawing software demonstrated that the UHMWPE occupies 57.9% of the femoral spacer (size #1, 57.9%) and 71.6% of the tibial (size #1, 71.6%) spacers. A reduced cement volume usage can provide better cost-effectiveness. However, major concerns exist with insufficient antibiotic elution concentration associated with lesser antibiotic-loaded cement usage. However, the current study results for the novel scaffold-like UHMWPE spacer have provided sufficient confidence that lesser cement volume usage may still provide clinically effective infection control capabilities with minimum gentamicin concentrations in the antibiotic-loaded cement group (1.9 μg/mL) remaining effective (>1 μg/mL) [29] at the end of the test period (30 days). However, tube dilution testing revealed less inhibitory capabilities against bacteria colonies including MRSA, CoNS and *E. coli*. The major reason may be the bone cement used in the current study, which was loaded with specific gentamicin concentrations that may not be appropriate antibiotic options or dosages against these pathogens. This reveals an important consideration that not all commercial preformed antibiotic-loaded cement spacers or their associated antibiotic formulations may fulfill ideal antibiotic options for infection control. Therefore, flexible antibiotic options (such as vancomycin and ceftazidime [7]) are of critical importance in clinical practice. 

There were several limitations with the current study:

- Concern of Bacterial Biofilm

The formation of bacterial biofilm is an important concern when foreign orthopedic devices are implanted [42], especially for the attachment on polyethylene material [43]. In the standard surgical procedure, an extensive debridement including removal of prosthesis and total synovectomy will be done before the implantation of spacers. Therefore, the majority of biofilm will be removed after the extensive debridement. In daily orthopedic practice, high dose antibiotic-loaded cement (more than 2 g antibiotic in 40 g PMMA powder) is routinely used for infection eradication. Therefore, the novel spacer loaded with high dose antibiotic-loaded cement will provide a high concentration antibiotic elution which will eradicate the bugs and reduce the change of bacteria to form biofilm. Further clinical trials will be required for demonstrating this opinion.

- *The Antibiotic-Loaded Cement Applied*

The current study has only considered a single antibiotic-loaded cement option for tibial spacer fatigue, wear, and antibiotic-elution testing. The application of the current spacer with other commercial antibiotic cements or surgeon-defined antibiotic formulations or dosages will have to be further validated for mechanical safety and biological efficacy concerns. In addition, the Palacos R + G (0.5 g gentamycin/40 g powder) is generally applied for prophylaxis use [44]. Due to limitations in availability, the Palacos R+G was utilized in the current study. For periprosthetic joint infection control, commercial product such as Copal G + C (1 g gentamycin + 1 g clindamycin), Copal G + V (0.5 g gentamycin + 2 g vancomycin), and VancoGenx (1 g gentamycin + 1 g vancomycin) are commonly utilized. In the current study, bone cement with lower dose of antibiotics and lesser surface area exposure for antibiotic-elution was applied. Even in this difficult condition the current spacer sets represented effective antibiotic elution concentration for infection control, which may enhance confidence before the device is sent for clinical trials.

- *Modified Test Condition*

The spacer is defined as a device used for temporary infection control and limb length (or soft tissue tension) maintenance. Generally, the recommended period for usage is 180 days under partial weight-bearing conditions. The load conditions in the current biomechanical test were modified with reference to an estimated partial weight-bearing condition [22]. These test results cannot represent patients who may be over-weight, subjected to full weight-bearing conditions without the utilization of assistive devices, or those performing high-strength activities post-implantation of the UHMWPE spacers.

- *Wear Rate Calculation*

Due to technical limitations, the weight or volumetric ratio of the UHMWPE and cement wear debris could not be identified. However, from wear pattern observations of the test specimens and SEM images for particle examination, most of the wear particle were determined to have originated from the UHMWPE material. Volumetric wear performance was then calculated by applying the appropriate density for UHMWPE.

- *Surface Condition and Material of Molding Device*

It was observed that the cement articulation was not ideally smooth. The major cause of this rough surface was the simple silicone mold was utilized for forming the articulating surface (Figure 3). The silicone mold material is soft and deformation of the cement during polymerization may occur (cement volume will expand but the mold was not strong enough to resist the expansion, so an irregular cement surface was then produced as shown in Figure 4). The wear test revealed acceptable results when comparing with the reported data in previous publication [24], even though the surface condition of the cement region was not ideal. However, the additional mechanical machining would not be recommended due to difficulty in clinical practice. The purpose of the polyethylene spacer was to prepare a ready-to-use solution with flexibility in antibiotics option, while surgeons are more familiar using a cement mold for geometrical formation. In addition, the performance of mechanical machining on the cement is not clear since it is a brittle polymer material. When using the cement with the polyethylene spacer in the current study, it becomes a composite structure which may exacerbate the difficulty in mechanical machining. We plan to improve the surface quality of the cement utilizing stainless steel with a polished female articulating surface feature to replace the use of silicone material for the mold in the future.

- *Unverified Canal Rod*

The canal rod in the current knee spacer set has only been introduced for its function and features but was not involved in the pre-clinical validation testing. Major safety and efficacy concerns exist with the utilization of the worst-case conditions, without the utilization of canal rod (wrapped with antibiotic cement). No fatigue testing was applied to the canal rods as they do not contribute to mechanical support. Antibiotic elution testing and wear testing were also not applied as the canal rods did not interfere with the antibiotic-loaded cement spacer and its articulation surface.

## 5. Conclusions

The scientific validation conducted in the current study with a novel scaffold-like polyethylene-based knee spacer design will provide surgeons with an improved knee joint infection control solution that has sufficient mechanical properties, anti-wear performance, and antibiotic elution. As a cement carrier, this novel device may offer surgeons more flexible antibiotic options and dosage adjustments for a more precise and effective infection control. Further clinical trials should be conducted to enhance clinical confidence regarding the use of this device in practical applications. 

## 6. Patents

TW M465151U  Porous artificial knee joint (patent of Taiwan, R.O.C.)
CN203341865U Porous type artificial knee joint (patent of Mainland China)

## Figures and Tables

**Table 1:** Geometrical information of femoral and tibial spacers.

	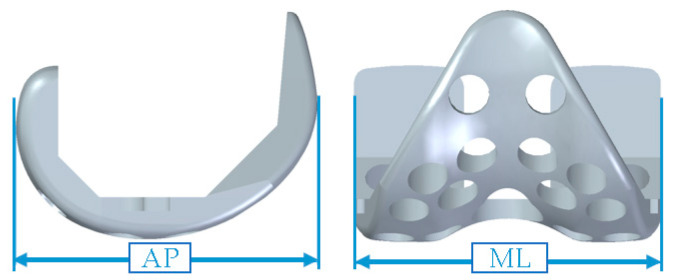		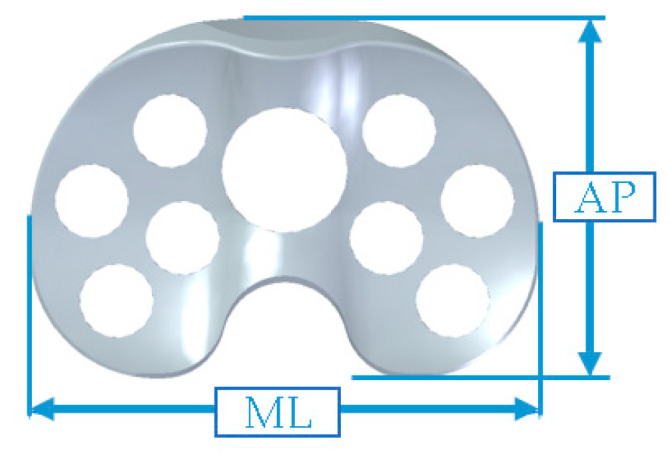	
**Size**	**Femoral Spacer**		**Tibial Spacer**	
	**AP (mm)**	**ML (mm)**	**AP (mm)**	**ML (mm)**
#1	56	56	42	63
#2	60	60	44.5	66
#3	64	64	47	69
#4	68	68	49.5	72
#5	72	72	52.5	76

**Table 2:** Wear testing load conditions.

Item	Condition
Test temperature	37 ± 2 °C
Flexion/extension	0° to 58°
Axial force	84 N to 1300 N
AP force	−265 N to 110 N
Axial torque	−1 Nm to 6 Nm

**Table 3:** Calculated wear rate (loss of dry weight) of the spacers.

	Loss of Dry Weight	
Specimen	Femoral Spacer (mg)	Tibial Spacer (mg)
#1	24.6	33.9
#2	16.1	3.51
#3	48.8	35.9
#4	−3.8*	7.6
#5	34.6	26.4
#6	47.6	5.37
Average	27.98 ± 20.12	18.78 ± 14.96
Average *	34.34 ± 14.25	18.78 ± 14.96
Commercial spacers [24]	149.6 ± 10	226.0 ± 88.3

* Negative value in weight loss was determined as bias and was removed from calculation.

**Table 4:** Morphological characteristics of wear particles.

Particle Material	Specimen	Equivalent Circle Diameter (μm)		Form Factor		Maximal Feret Diameter (μm)		Minimal Feret Diameter (μm)		Area (μm^2^)		Perimeter (μm)		Aspect Ratio		Particle Analyzed
		Mean	Range	Mean	Range	Mean	Range	Mean	Range	Mean	Range	Mean	Range	Mean	Range	
UHMWPE	#1	0.49	0.12–1.97	0.54	0.07–1.00	0.92	0.14–4.61	0.47	0.09–2.81	0.3	0.01–3.06	2.96	0.38–19.36	1.85	1.05–5.65	105
	#2	0.45	0.13–1.52	0.56	0.07–1.00	0.81	0.16–3.17	0.45	0.11–2.44	0.24	0.01–1.82	2.67	0.41–13.87	1.74	1.07–3.10	109
	#3	0.49	0.11–1.79	0.50	0.07–1.00	0.95	0.14–4.92	0.47	0.09–2.01	0.3	0.01–2.51	3.24	0.36–20.75	1.98	1.06–5.00	105
	#4	0.54	0.11–2.13	0.46	0.06–0.99	1.04	0.14–5.14	0.54	0.11–2.05	0.32	0.01–3.56	3.59	0.38–24.10	1.93	1.08–5.03	87
	#5	0.46	0.13–1.95	0.52	0.06–1.00	0.81	0.18–3.08	0.45	0.11–2.21	0.26	0.01–3.00	2.75	0.46–15.82	1.87	1.03–5.36	99
	#6	0.54	0.13–1.64	0.42	0.05–1.00	1.11	0.18–3.69	0.56	0.11–1.77	0.32	0.01–2.11	3.87	0.44–13.51	2.00	1.08–5.61	93
	**Average**	**0.50 ± 0.04**		**0.50 ± 0.05**		**0.94 ± 0.12**		**0.49 ± 0.05**		**0.29 ± 0.03**		**3.18 ± 0.48**		**1.89 ± 0.10**		**-**
PMMA	#1	0.42	0.36–0.49	0.75	0.70–0.82	0.52	0.45–0.59	0.41	0.34–0.47	0.14	0.10–0.19	1.55	1.32–1.85	1.24	1.17–1.29	3
	#2	0.39	0.30–0.48	0.81	0.71–0.88	0.50	0.37–0.55	0.36	0.30–0.47	0.12	0.07–0.18	1.37	1.07–1.60	1.37	1.10–1.85	4
	#3	0.68	0.29–0.90	0.57	0.42–0.72	1.00	0.52–1.25	0.62	0.22–0.83	0.4	0.07–0.64	2.86	1.26–3.79	1.72	1.45–2.38	4
	#4	0.37	0.15–0.61	0.84	0.78–0.94	0.46	0.18–0.74	0.35	0.15–0.56	0.14	0.02–0.29	1.30	0.49–2.16	1.24	1.14–1.30	3
	#5	N/A	N/A	N/A	N/A	N/A	N/A	N/A	N/A	N/A	N/A	N/A	N/A	N/A	N/A	0
	#6	0.26	0.17–0.40	0.84	0.70–0.93	0.35	0.24–0.55	0.22	0.13–0.32	0.06	0.02–0.13	0.89	0.57–1.38	1.61	1.19–1.84	4
	**Average**	**0.42 ± 0.15**		**0.76 ± 0.12**		**0.57 ± 0.25**		**0.39–0.15**		**0.17 ± 0.13**		**1.59 ± 0.75**		**1.43 ± 0.22**		**-**
